# Reference Values of the Forearm Tremor Power Spectra for Youth Athletes

**DOI:** 10.5114/jhk/159644

**Published:** 2023-01-20

**Authors:** Jan Gajewski, Joanna Mazur-Różycka, Michał Górski, Krzysztof Buśko

**Affiliations:** 1 Chair of Human Biology, Józef Piłsudski University of Physical Education in Warsaw, Warsaw, Poland.; 2 Department of Ergonomics, Central Institute for Labour Protection-National Research Institute, Warsaw, Poland.; 3 Department of Kinesiology, Institute of Sport-National Research Institute, Warsaw, Poland.; 4 Institute of Physical Culture, Kazimierz Wielki University, Bydgoszcz, Poland.

**Keywords:** sports, oscillations, accelerometry, resonance

## Abstract

The aim of the study was to determine reference graphs of power spectral density functions of forearm physiological tremor and to compare their parameters in the male and female population of young athletes from various sports. One hundred fifty-nine (159) female (15.7 ± 2.1 years, 59.8 ± 8.1 kg, 169.1 ± 7.5 cm) and 276 male (16.4 ± 1.9 years 72.7 ± 10.3 kg and 180.9 ± 8.7 cm) youth athletes participated in the study. Forearm tremor was measured accelerometrically in a sitting position. Power spectrum density (PSD) function was calculated for each individual tremor waveform. Because of right skewness of power distribution, the PSD functions were subjected to logarithmic transformation. Average log-powers in low (2–4 Hz) and high (8–14 Hz) frequency ranges and mean frequencies in those ranges were analyzed. Tremor log-powers for male were greater than for female athletes (p < 0.001), while frequencies of spectrum maxima did not differ from each other. Frequencies of spectrum maxima correlated (p < 0.001) with age (r = 0.277 and 0.326 for males and females, respectively). The obtained reference functions may be utilized in order to quantify and assess tremor size and its changes evoked by stress and fatigue, which can be applied for selection and training monitoring in sports, but also in medicine for detection and diagnosis of pathologic tremor in young individuals.

## Introduction

Many professions require accuracy of movement. Similar expectations may concern surgeons or pistol shooters. The present study deals with tremor in youth athletes. It seems reasonable to know how the values of tremor parameters are distributed among athletes, especially when thinking of fatigue diagnostics or selection for different sports.

Physiological tremor is defined in the literature as involuntary and continuous oscillation of the whole body or its part observed in healthy subjects (McAuley and Marsden, 2000). Physiological tremor is produced by interaction of mechanical and neural factors (Nagamori et al., 2018). Postural tremor occurs when a subject tries to maintain a motionless position of the limb acting against gravity (Takanokura and Sakamoto, 2005). Force fluctuations are caused by summing of single motor units’ activities (Lodha and Christou, 2017), the stretch reflex responses and synchronized firing of motor units (Christakos et al., 2006; Yoo et al., 2019), as well as rhythmic activity in the central nervous system (Gandevia, 2001). Main mechanical factors influencing tremor include limb inertia and visco-elastic properties of muscles (Lakie et al., 2012). Irregularities in force production generate mechanical resonance (Carignan et al., 2012), frequency of which is related to muscle stiffness and limb size. The greater the muscle stiffness and the lower the limb inertia, the greater is the resonant frequency. Resonant oscillations are observed, called the load-dependent maximum of the tremor spectrum. Its frequency is 3–4 Hz for a forearm (Viitasalo and Gajewski, 1994), 7–10 Hz for a hand (Héroux et al., 2009) and about 15–30 Hz for a finger (Aljihmani et al., 2019). Probably, the low frequency maximum arises as a result of interaction of the active (stretch reflex) and passive resonance properties of the limb (Nagamori et al., 2018; Takanokura and Sakamoto, 2001). However, Lakie et al. (2012) did not find any link between the low frequency tremor and the stretch reflex.

The tremor spectrum also includes a “physiological component” with a frequency of about 10 Hz. It is assumed that synchronization of motor units firing at this frequency may be caused by oscillations in the stretch reflex loop (Nagamori et al., 2018; Miao and Sakamoto, 1995, 1997). McAuley and Marsden (2000) believe that these oscillations result from delay in nerve signal conduction. Other researchers (Lakie et al., 2012) see the reasons for the formation of the “physiological component” in the rhythmic activity of the motor cortex. The tremor components in the 8–12 Hz range do not change their frequency when the external load changes.

The tremor amplitude varies greatly between individuals (Lakie et al., 2012). However, the power distribution within the frequency domain (shape of the power spectral density function – PSD) is similar for different individuals (Raethjen et al., 2002). The parameters that do not significantly differentiate between individuals are frequencies of the maxima and the frequency range (McAuley and Marsden, 2000). The shape of the PSD function largely depends on the measurement conditions. Inertia and stiffness of the external load (Miao and Sakamoto, 1995; Takanokura and Sakamoto, 2005), as well as the position of the tested limb influence tremor amplitude and its distribution along the frequency domain.

Parameters of physiological tremor are affected by physical exertion (D’Addona et al., 2014; Gajewski, 2006) and emotions (Klein et al., 2020). In both cases increased tremor amplitude reflects the state of fatigue or anxiety and might be used as their quantitative, non-invasive indicator.

The distribution of the signal power in the frequency domain allows to assess tremor amplitude as well as the characteristic frequencies. In addition, for a more accurate description of the phenomenon, it would be advisable to investigate the relationship between the variables describing tremor and the gender, age and basic anthropometric characteristics of the subjects. Another issue is to examine the statistical distributions of the variables studied in the study population.

Therefore, the aim of the present study was to determine reference values of power spectral density functions of forearm physiological tremor and to compare their parameters in the male and female population of youth athletes from various sports.

## Methods

### Participants

The research was conducted with the consent of the Senate Ethics Committee at the Józef Piłsudski University of Physical Education in Warsaw, Poland (no. SKE 01-27/2011). All procedures were carried out according to the Declaration of Helsinki. The tested athletes were informed about the purpose and methods of the research and about the possibility of withdrawing from the research at any stage. Athletes gave their written consent to participate in the study. Before the tests, each examined person underwent a medical examination. None of the participants had any neurological disease that could affect the tremor measurements.

The group consisted of 435 youth athletes, including 159 women and 276 men, practicing various sports. The tested athletes belonged to the national team of juniors or the national team of seniors and represented a very high sports level: Medalists of the Polish Junior and Senior Championships, the World and European Championships. Somatic characteristics of participants are presented in Table 1. The 'Other' category includes handball, judo and weight lifting for men and badminton, horse riding, weight lifting and table tennis for women.

**Table 1:** Somatic characteristics of the tested female (n = 159) and male (n = 276) athletes.

	Sport discipline	Age [years]	Body mass [kg]	Body height [cm]	Training experience [years]
Women	Canoeing (n = 13)	16.5 ± 1.3	60.6 ± 6.1	168.5 ± 6.3	6.2 ± 1.9
	Swimming (n = 93)	14.6 ± 1.3	58.4 ± 7.0	169.1 ± 6.0	6.8 ± 2.1
	Volleyball (n = 11)	16.6 ± 1.0	69.0 ± 7.5	183.5 ± 7.0	5.6 ± 2.2
	Wrestling (n = 14)	19.4 ± 1.7	60.7 ± 7.8	164.9 ± 5.6	8.4 ± 1.7
	Other (n = 28)	18.7 ± 4.4	61.3 ± 10.2	165.8 ± 7.7	7.9 ± 2.5
	Total (n = 159)	15.7 ± 2.1	59.8 ± 8.1	169.1 ±7.5	7.0 ± 2.3
Men	Canoeing (n = 30)	17.5 ± 1.9	73.1 ± 7.9	180.4 ± 6.0	7.0 ± 3.9
	Swimming (n = 102)	14.5 ± 2.4	67.0 ± 7.1	179.5 ± 5.8	7.1 ± 2.5
	Volleyball (n = 29)	17.2 ± 0.9	84.9 ± 7.0	196.7 ± 5.4	6.4 ± 2.3
	Wrestling (n = 23)	19.3 ± 1.9	72.6 ± 11.4	171.8 ± 7.5	8.3 ± 2.9
	Baseball (n = 16)	23.9 ± 2.6	87.1 ± 11.0	181.8 ± 7.5	9.1 ± 1.1
	Ice hockey (n = 47)	17.3 ± 0.8	75.9 ± 7.0	179.8 ± 5.9	9.6 ± 1.1
	Archery (n = 19)	19.1 ± 5.1	72.0 ± 10.8	177.3 ± 7.5	6.5 ± 4.1
	Other (n = 40)	20.8 ± 6.8	77.5 ± 11.5	181.4 ± 6.7	7.7 ± 2.9
	Total (n = 276)	16.4 ± 1.9	72.7 ± 10.3	180.9 ± 8.7	7.7 ± 3.0

### Physiological Tremor Measurements

Forearm tremor was measured accelerometrically between 9.00 and 12.00 AM. During measurements, the participant was sitting on a chair with their torso and elbow supported. Their horizontally aligned forearm was kept in a supine position and held as stationary as possible. The accelerometer was attached to a one-kilogram weight placed on the participant's hand. The accelerometer was positioned over the participant’s wrist. The 1 kg inertial load (Tomczak et al., 2014) did not exceed 10% of the elbow flexors MVC (maximum voluntary contraction), even in potentially weaker participants. Therefore, it was assumed that the external load applied did not cause fatigue that could affect physiological tremor. The 31.72-s waveform of the vertical component of acceleration was recorded using the ZPP-3D/BC Acceleration Measurement Set (JBA Zb. Staniak, Poland).

### Frequency Analysis

Sampling was performed with a frequency of 200 Hz. The acceleration signal, before analog-to-digital conversion, was low-pass filtered at Nyquist’s frequency (100 Hz) in order to eliminate the ‘mirror effect’. Then the signal registered was subjected to frequency analysis, the purpose of which was to obtain the power spectral density (PSD) function. This function describes the distribution of the signal variance in the frequency domain. The entire set of 6144 samples was divided into six segments of 1024 samples each. The fast Fourier transform (FFT) procedure (MATLAB R2007a) was used for PSD estimation for each segment. The cosine window was applied. The resultant power spectral density function (PSD) for the single registered signal was calculated as the average of PSDs obtained for each segment.

The indices describing the power and frequency of the tremor signal were calculated for each participant based on the power spectral density (PSD) function.

The following variables were defined for the further analysis: Log amplitude indicator defined as an average of logarithms of power components from f1 to f2 range:


$$L(f_{1},f_{2})=\frac{1}{f_{2}-f_{1}}\int_{f_{1}}^{f_{2}}InPSD(f)df,\text{ (1)}$$

where:

L(f_1_,f_2_) – log amplitude indicator of frequency band between f_1_ and f_2_,

PSD(f) – power spectral density function,

f_1_, f_2_ – the boundaries of the frequency band.

Mean frequency of power components from f1 to f2 range defined as:


$$F(f_{1},f_{2})=\frac{\int_{f_{1}}^{f_{2}}f\cdot PSD(f)df}{\int_{f_{1}}^{f_{2}}PSD(f)df'}\text{ (2)}$$

where:

F(f_1_,f_2_) – mean frequency power,

PSD(f) – power spectral density function,

f_1_, f_2_ – the boundaries of the frequency band.

### Statistical Analysis

Statistical analysis was performed using Statistica 13.0 (TIBCO Software Inc., 2017) or Microsoft Excel. The normality of the distributions of the variables describing tremor spectra was determined using the Kolmogorov-Smirnov test (*p* > 0.20). Comparisons of means between male and female groups were done using the Hotelling T square test. Effect sizes were estimated using Hedges’ g. Correlations between tremor indicators and the basic anthropological variables (body weight, body height, BMI and age) were determined using the Spearman correlation. The Pearson correlation coefficient was used to assess the relationship between the low- and high-frequency components of tremor signal power with average frequencies. The level of significance was set at α = 0.05.

## Results

The tremor PSD function was defined from 0 to 100 Hz. However, it was considered that the components with frequencies less than 1 Hz were the result of slow, controlled movements and this range was not included in further analysis. As it was calculated, the components from the range 1 to 25 Hz represented on average 97% and 98% of the remaining signal power (variance) for women and men, respectively. Therefore, it was assumed that further analyses would concern the components of tremor in the range from 1 to 25 Hz.

In order to initially test the normality of the distribution of individual components of the tremor signal power in the studied groups, the value of the skewness coefficient (S) was calculated for each of the PSD components along the frequency domain. The S(f) functions were obtained in this way for female and male groups. The values of the function S(f) for the analysed frequencies ranged from 2 to 16, which indicated a clear right-skewness of the distributions of the power spectrum components. After the logarithmic transformation, the skewness frequency series obtained for the distributions of the logPSD(f) functions showed only a small, acceptable deviation from zero, which proves the relative symmetry of the distributions tested.

It was considered that variables describing power or the amplitude of the tremor signal should be computed from the logarithms of the power density function. The average power density function along with the frequency series corresponding to standard deviations were calculated according to the following formulas:


$$PSD_{a}(f)=\exp\left[\frac{1}{n}\sum_{i=1}^{n}In(PSD_{i}(f))\right],\text{ (3)}$$

$$PSD^{\pm }(f)=\exp\left[InPSD_{a}(f)\pm SD(InPSD(f))\right],\text{ (4)}$$

where:

PSD_a_(f) – average power density function,

PSD^±^(f) – frequency series corresponding to standard deviations of the power density function,

n – number of subjects,

i = 1, 2, … n,

SD(lnPSD(f)) – standard deviation of lnPSD for the component of the frequency f.

**Figure 1:**
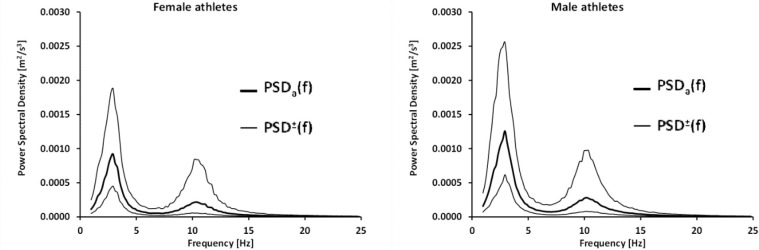
Mean (± SD) power spectrum density function of the forearm tremor for female athletes (n = 159) (left) and male athletes (n = 276) (right). PSD_a_(f) – average power density PSD^±^(f) – frequency series corresponding to standard deviations of the power density function.

The averaged power density function of the tremor signals for women and men obtained following this method is presented in Figure 1.

**Figure 2:**
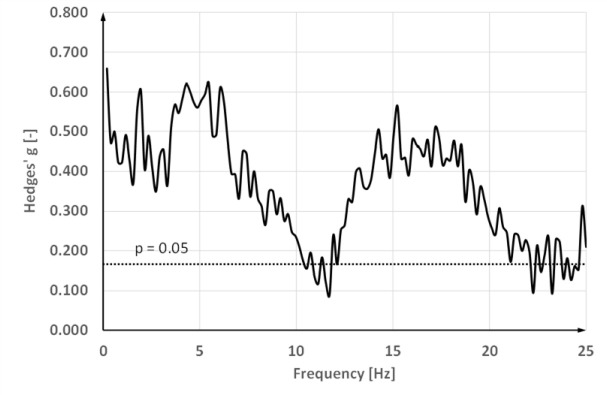
The Hedges g function evaluating the effect size of the difference in tremor spectra for men and women in the frequency domain.

It can be noted that the values of power for women are less than for men. Figure 2 shows the effect size against the frequency. The Hedges g values for log power differences are presented as a function of frequency. The level of significance is marked with a dotted, horizontal line.

Descriptive characteristics of variables describing the signal log-power (logarithmic amplitude indicators), as well as mean frequencies in selected frequency ranges are presented in Table 2. The frequency range of 10 to 20 Hz has previously been reported to be the most sensitive to fatigue (Viitasalo and Gajewski, 1994). The ranges of 2–5 Hz and 8–14 Hz are related to the location of the spectrum peaks.

**Table 2:** Means (± SD) of variables describing tremor log-power (logarithmic amplitude indicators L) as well as mean tremor frequencies (F) in selected frequency ranges for male and female athletes.

	Women (n = 159)	Men (n = 276)	Hedges’ g
L(10, 20) [-]	−10.25 ± 0.87	−9.91 ± 0.73***	0.410
L(2, 4) [-]	−7.50 ± 0.55	−7.14 ± 0.59***	0.640
L(8, 14) [-]	−9.19 ± 0.95	−8.90 ± 0.83***	0.325
L(1, 25) [-]	−10.08 ± 0.69	−9.76 ± 0.62***	0.484
F(10, 20) [Hz]	12.27 ± 0.69	12.36 ± 0.70	0.130
F(2, 4) [Hz]	2.92 ± 0.14	2.93 ± 0.15	0.008
F(8, 14) [Hz]	10.59 ± 0.47	10.55 ± 0.52	0.078
F(1, 25) [Hz]	6.12 ± 1.62	5.98 ± 1.29	0.096

*** p < 0.001

It is worth noting that variables describing the power of tremor (L(fi, fj)) differ significantly in the male and female groups, while for the mean frequencies (F(fi, fj)), despite the relatively large number of participants, no such differences were detected. The distributions of logarithmic indicators of tremor amplitude and distributions of mean frequencies were proved to be normal (Kolmogorov-Smirnov test, *p* > 0.20).

In order to find out whether the power components concentrated around the low- and high-frequency maxima are related to each other, and whether they are related to the mean frequencies, the correlation coefficients between the variables describing the spectrum shape were calculated for both groups.

Due to the relatively large number of participants, the substantive effect (r^2^ > 10%) occurred in both groups in the event of the relationship L(2, 4) and L(8, 14): r = 0.436 (*p* < 0.001) and r = 0.531 (*p* < 0.001), for women and men, respectively. This proves a clear relationship between the power of low- and high-frequency tremor components.

The relationships of logarithmic indicators of tremor amplitude and average frequencies in the range of maxima with the basic anthropometric variables (body weight, height and BMI) are presented in Table 3. The analysis was performed on the basis of Spearman's correlation coefficients due to the deviations of anthropometric variables from the normal distribution.

**Table 3:** Spearman's correlation coefficients between tremor indices and anthropometric variables for women (n = 159) and men (n = 276).

		Body mass	Body height	BMI	Age
Women	L(2, 4)	−0.023	−0.075	0.047	−0.198
	L(8, 14)	−0.144	0.022	−0.194	−0.289**
	F(2, 4)	0.088	−0.056	0.154	0.277**
	F(8, 14)	−0.082	−0.225	0.077	0.101
Men	L(2, 4)	0.047	0.036	0.000	−0.213**
	L(8, 14)	−0.049	0.011	−0.094	−0.172
	F(2, 4)	0.160	−0.077	0.264***	0.326***
	F(8, 14)	0.020	−0.109	0.139	0.145

*** p < 0.001, ** p < 0.01 (Bonferroni criterion considered)

It was found that neither body weight nor height showed any significant relationship with the parameters of tremor. There is a significant correlation that repeats in both groups between the average frequency in the range (2–4 Hz), which corresponds to the low-frequency maximum, and the calendar age. The older the respondents were, the higher was the frequency.

The standardized tremor log power (LPSD^*^(f)) for the selected person can be obtained on the basis of their lnPSD (f) function and the mean (lnPSDa(f)) and standard deviation (SD(f)) functions:


$$LPSD_{i}^{*}(f)=\frac{InPSD_{i}(f)-InPSD_{a}(f)}{SD(f)},\text{ (6)}$$

where:

$LPSD_{i}^{*}$ (f) – standardized tremor log power for the i-th person,

PSD_a_(f) – power spectral density function for the ith person,

PSD_a_(f) – average power density function,

SD(f) – log power standard deviation.

**Figure 3:**
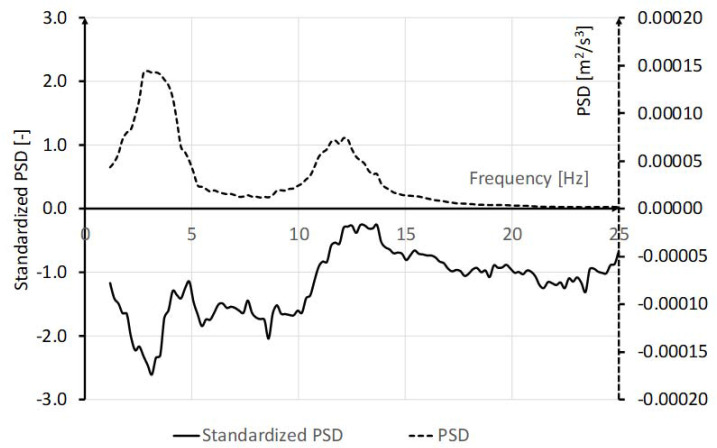
Example of the standardized power density function of the tremor signal registered for a female pistol shooter.

The practical usefulness of the analysis of the standardized PSD is presented in Figure 3. The chart presents the standardized tremor spectrum processed for a female pistol shooter (21 years, 56 kg, 167 cm) who was tested for another study. A well-pronounced minimum at the frequency of about 2.5 Hz is clearly seen, indicating an ability to control tremor amplitude and reduce its values at resonance frequencies. Extremely small values of the standardized power at the minimum were below the third percentile. Standardized PSD values for high frequency tremor were greater and placed slightly below average.

## Discussion

The aim of the research was to determine the reference spectral density function of tremor signals for the population of youth athletes. A total of 435 people were examined. Previous studies have not reported the results of accelerometric measurements of this number of healthy young people. Several population studies have been conducted, but they concerned qualitative and questionnaire data analysis (Louis et al., 2019). Those studies aimed mainly to assess the essential tremor or Parkinson’s disease prevalence (de Araújo et al., 2020). Elble et al. (2006) demonstrated that tremor amplitude was logarithmically related to a 5-point rating scale. Those authors did not analyze distribution of tremor amplitude (or power) in the frequency domain. The results of our study confirm those findings. However, reports on the logarithmic nature of tremor size had appeared even earlier (Louis et al., 2001; Viitasalo and Gajewski, 1994). Our results provide evidence that tremor amplitude or power is log-normally distributed among the population. In consequence tremor amplitude of the standardized value equal to 2 (ca. 97^th^ percentile) is about five times greater than the average in the low frequency range (2–5 Hz), whereas for the high frequency range (8–14 Hz), it exceeds even fifteen times the average value. In the light of these findings, some cases of essential tremor might be considered as physiological ones, especially that essential tremor is not associated with other neurological signs, such as dystonia, ataxia, or parkinsonism (Bhatia et al., 2018). Such differences in tremor amplitude in humans make changes in tremor, such as exercise induced, difficult to quantify and appear unrelated to the amount of exertion involved. The logarithmic transformation enables quantitative analysis of tremor and its changes under conditions of fatigue or stress (Gajewski, 2006).

The present paper presents the reference power spectral density functions of the tremor signal for young women and men practicing various sports based on the recorded individual tremor waveforms. The obtained reference functions were characterized by qualitative similarity. Both for women and men, they had a similar shape and proportions, and a similar position of the peaks. According to the results of other authors (Raethjen et al., 2002; Tomczak et al., 2014), these maxima occurred at frequencies of approx. 3 Hz and 10 Hz. The component in the 10 Hz range was first described in 1886 by Schäfer and is referred to in the literature as “the physiological component”. Components with frequencies greater than 25 Hz were excluded from the analysis. It was assumed that they had no practical significance and reflected the cushioning properties of the palm surface.

The shape of the spectrum may be influenced by the inertial load (Miao and Sakamoto, 1995). Since the low-frequency peak of the spectrum is the result of mechanical resonance (Vernooij et al., 2013), the position of the maximum depends on the moment of inertia of the forearm and muscle stiffness. In our research, an inertial load with a one-kilogram weight placed on the palm part of the hand was used. Certainly the resultant elbow torque did not engage more than 10% of the maximum strength in the elbow joint. Some authors believe that the amplitude increases with an increasing load (McAuley and Marsden, 2000). However, tremor power for both lower and higher frequencies was less for girls than boys, which means that the applied load was not too high for women, who were potentially weaker than men.

Interestingly, no correlation between variables describing the amplitude and frequency of the tremor maxima and the body weight and height was found. Assuming that these anthropometric variables are directly related to the moments of inertia of the forearm, one could expect that people with a larger body size will experience resonance at lower frequencies. Of course, muscle stiffness should also be considered. It can be assumed that the level of stiffness will be higher at a greater cross-section of the muscles and smaller at greater muscle length. If we accept the opinion that the stretch reflex is not involved in the generation of tremors (Vernooij et al., 2013), it should be assumed that in people with a large body size, muscle stiffness is greater. It can also be considered that the resultant stiffness does not result only from the passive properties of the muscle tissue. The stretch reflex can also be seen as a phenomenon that actively changes the resultant stiffness (Yoo et al., 2019). Takanokura and Sakamoto (2001) presented a model showing that sensitivity of the stretch reflex to the changes of muscle length directly determines the resultant muscle stiffness.

The observed positive correlation of the frequency of the resonant peak and the age of the athletes is surprising. It has been shown that among older subjects, the tremor peaks occur at higher frequencies. It is unclear whether this is an effect directly related to age or rather to selection mechanisms in sport. It is hypothesized that longer training experience could influence muscle stiffness (an increased cross-sectional area of muscles).

The ability to limit the amplitude of involuntary movements is crucial in many sports such as shooting, archery, etc. Tremor measurement results compared to the presented reference values of the tremor power spectrum provide the basis to indicate the individuals distinguished by small tremor amplitude and predisposed to these sports. The example of the pistol shooter presented in this study leads to the conclusion that specific training can eliminate low frequency force fluctuation. According to the findings of Vernooij et al. (2013), reducing resonant oscillation may be achieved by the minimization of the neural voluntary drive.

Fatigue diagnosis is one of the important issues of sports training (Tsarbou et al., 2021). The tremor spectrum changes significantly due to fatigue. Standardizing these changes based on reference values would create the possibility of making quantitative and non-invasive assessments of fatigue.

The authors realize that there are limitations to the present study. First, the subjects were not randomly selected and represented various sports disciplines. Particular sport groups were of different sizes. The authors were not able to provide the subjects with exactly the same conditions related to mental stress and physical exertion in the time preceding the measurements. Stress and fatigue can increase tremor amplitude (Aljihmani et al., 2019; Growdon et al., 2000). Nevertheless, in the authors’ opinion the obtained forearm tremor spectral density graphs could serve as comparative data for tremor measurements in youth athletes.

Results from all subjects are presented together, although the authors recognize that the type of physical activity practiced may be related to tremor characteristics. It was found that, in men, tremor amplitude in archers was smaller than in volleyball players and swimmers (as in women). The theme of tremor in archers is confirmed in the literature (Ogasawara et al., 2021), which, as in shooters (Mon-López et al., 2019), is related to the specificity of the discipline and correlates with sports performance.

## Conclusions

It was concluded that in view of the clear right-skewness of the distributions of tremor amplitude and power, all variables related to tremor size should undergo logarithmic transformation before quantitative analysis. The obtained reference functions may be utilized in order to quantify and assess tremor magnitude and its changes evoked by stress and fatigue, which can be applied for the selection and monitoring of training in sports. The standardized courses of tremor spectra may be applied in medicine for detection and diagnosis of pathologic tremors in young individuals.
